# Diagnostic accuracy for major depression in multiple sclerosis using self-report questionnaires

**DOI:** 10.1002/brb3.365

**Published:** 2015-07-14

**Authors:** Anja Fischer, Marcus Fischer, Robert A Nicholls, Stephanie Lau, Jana Poettgen, Kostas Patas, Christoph Heesen, Stefan M Gold

**Affiliations:** 1 Department of Health Psychology, King’s College LondonSE1 9RT, London, UK; 2 Center for Molecular Neurobiology, Institute of Neuroimmunology and Multiple Sclerosis (INIMS), University Medical Center Hamburg-EppendorfHamburg, Germany; 3 Department of Pharmaceutical Chemistry, University of California San FranciscoSan Francisco, California, 94158; 4 Structural Studies Division, MRC Laboratory of Molecular BiologyCambridge, CB2 0QH, UK; 5 Department of Neurology, University Medical Center Hamburg-EppendorfHamburg, Germany; 6 Department of Medical Psychology, University Medical Center Hamburg-EppendorfHamburg, Germany; 7 Department of Psychiatry and Psychotherapy, Campus Benjamin Franklin (CBF), Charité UniversitätsmedizinBerlin, Germany

**Keywords:** Major depressive disorder, multiple sclerosis, self report, sensitivity and specificity, validation studies

## Abstract

**Objective:**

Multiple sclerosis and major depressive disorder frequently co-occur but depression often remains undiagnosed in this population. Self-rated depression questionnaires are a good option where clinician-based standardized diagnostics are not feasible. However, there is a paucity of data on diagnostic accuracy of self-report measures for depression in multiple sclerosis (MS). Moreover, head-to-head comparisons of common questionnaires are largely lacking. This could be particularly relevant for high-risk patients with depressive symptoms. Here, we compare the diagnostic accuracy of the Beck Depression Inventory (BDI) and 30-item version of the Inventory of Depressive Symptomatology Self-Rated (IDS-SR_30_) for major depressive disorder (MSS) against diagnosis by a structured clinical interview.

**Methods:**

Patients reporting depressive symptoms completed the BDI, the IDS-SR_30_ and underwent diagnostic assessment (Mini International Neuropsychiatric Interview, M.I.N.I.). Receiver-Operating Characteristic analyses were performed, providing error estimates and false-positive/negative rates of suggested thresholds.

**Results:**

Data from *n* = 31 MS patients were available. BDI and IDS-SR_30_ total score were significantly correlated (*r* = 0.82). The IDS-SR_30_total score, cognitive subscore, and BDI showed excellent to good accuracy (area under the curve (AUC) 0.86, 0.91, and 0.85, respectively).

**Conclusion:**

Both the IDS-SR_30_ and the BDI are useful to quantify depressive symptoms showing good sensitivity and specificity. The IDS-SR_30_ cognitive subscale may be useful as a screening tool and to quantify affective/cognitive depressive symptomatology.

## Significant Findings

1. IDS-SR_30_ and the BDI are valid measures for multiple sclerosis-associated major depression.
2. The IDS-SR_30_ cognitive subscale may be suitable as a screening tool in MS depression.
3. The IDS-SR_30_ covers all diagnostic criteria of MDD and is available in numerous languages and free of charge, making it a particularly useful tool for depression screening in MS.

## Limitations

1. Small sample size
2. Depression self-report questionnaires are not suitable for distinction between *different affective disorders*.

## Introduction

Multiple sclerosis (MS) is an inflammatory, demyelinating disease of the central nervous system and is regularly accompanied by psychiatric symptoms such as depression (Feinstein et al. 2014). With a lifetime risk of up to 50% and a point prevalence of up to 25%, major depressive disorder (MDD) is a frequent comorbidity of MS (Patten et al. 2003). Multiple sclerosis-associated depression has a substantial negative impact on patients’ quality of life, cognition, and psychosocial functioning (Hakim et al. 2000; Sa 2008). Higher levels of depressive symptoms are also linked to poorer treatment compliance (Ivanova et al. 2012), and thus can affect long-term health outcomes. If left untreated, depressive symptoms in MS may worsen over time (Ensari et al. 2014). Despite the high clinical relevance of depression in MS, it remains frequently underdiagnosed and undertreated.

The diagnostic criteria for MDD include a number of somatic and vegetative symptoms that overlap with typical symptoms of MS (e.g., fatigue, sleep disturbance, impaired concentration), which can make accurate MDD diagnosis particularly difficult in this patient population. Therefore, valid and reliable, easy-to-use diagnostic tools taking into account the potential confounding of MS symptoms are needed. Adjustment of cutoff scores may be required to prevent false diagnoses due to somatic-symptom-related score inflation. This is particularly important in patients who might be at risk for a comorbid mood disorder, for example, patients with elevated self-reported depressive symptoms.

A wide range of self-rated questionnaires are available for quantification of depression. Some of these have been validated and used in MS patients (see Avasarala et al. 2003; Benedict et al. 2003; Moran and Mohr 2005; Mohr et al. 2007; Honarmand and Feinstein 2009; Quaranta et al. 2012). Guidelines published by the American Academy of Neurology recommended only the BDI as well as a two-question tool to screen for depressive disorders with a weak level of evidence and did not find sufficient evidence for other instruments (Minden et al. 2014).

Importantly, only a few studies to date (Sullivan et al. 1995; Pandya et al. 2005; Honarmand and Feinstein 2009; Quaranta et al. 2012; Patten et al. 2015) have used a structured clinical interview to establish MDD diagnosis, and only the most recent ones also included Receiver-Operating characteristics (ROC) analysis, the gold standard to verify diagnostic accuracy. The Hospital Anxiety and Depression Scale (HADS) showed good diagnostic accuracy (Honarmand and Feinstein 2009), however, it only covers some of the diagnostic criteria of MDD. Moreover, it is copyrighted and may not be easily available, particularly for clinics or research groups in developing countries. A clinician-based, MS-specific depression scale (MSDRS (Quaranta et al. 2012)) also achieved good accuracy overall, however, it has relatively poor sensitivity (38%) and so far has only been used in Italian patients. Finally, a very recent paper demonstrated good accuracy of the patient health questionnaire PHQ-9, the Center for Epidemiologic Studies Depression rating scale (CES-D), and the HADS in MS (Patten et al. 2015). However, there is still paucity of data directly comparing different self-report questionnaires head-to-head and against structured interviews. No study to date has addressed this question in German-speaking MS patients.

The 30-item self-rated Inventory of Depressive Symptomatology (IDS-SR_30_) was developed as part of the STAR*D trial (Rush et al. 1996) and has been validated for several patient populations with physical illness so far but not for MS. In contrast to most self-rated questionnaires for depression such as the Beck Depression Inventory (BDI) or the HADS, it assesses all symptom domains for MDD as designated in the DSM-IV and is available both in patient self-rating as well as clinician-based rating form. Moreover, it has been validated in more than 30 languages and is freely available (http://www.ids-qids.org/) without licensing charges. It also offers a self-rated validated 16-item short version (QIDS-SR) and subscales providing separate scores for cognitive and somatic symptoms that have been derived (Duivis et al. 2013). It might therefore be a promising tool to screen for and quantify depressive symptomatology in MS.

## Aims of the Study

Here, we compare diagnostic accuracy of the BDI, the IDS, its subscales, and its short form (QIDS) in a group of German MS patients who reported elevated depressive symptoms. This sample might therefore model a clinical situation where detection of MDD is particularly important. We aim to establish meaningful threshold values based on a structured clinical interview.

## Methods

### Subjects

MS patients (*n* = 31) were recruited via the MS clinic of the University Medical Center Hamburg Eppendorf using our patient database and written consent prior to inclusion in the study was obtained. We contacted patients by mail if the scores from their last clinical visit recorded in the database indicated elevated depressive symptoms as measured by the Mood subscale of the Hamburg Quality of Life Questionnaire for MS (HAQUAMS) (Gold et al. 2001).

### Diagnosis of major depression

Patients underwent structured diagnostic interviews by trained raters (A.F., S.L.) (The Mini International Neuropsychiatric Interview, M.I.N.I.) (Ackenheil et al. 1999). Several approaches have been proposed to implement DSM-IV diagnostic criteria in patients with physical illnesses: “aetiological” (case-by-case exclusion of somatic symptoms judged likely to be due to the comorbid medical illness), “inclusive” (use all symptoms regardless of etiology), and “substitutive” (substitution of most or all somatic symptoms with additional cognitive or affective symptoms). For the current study, we used the inclusive approach, that is, MDD diagnosis was made if a patient met at least five of the nine criteria which must include “depressed mood” or “loss of interest/anhedonia.”

### Self-report measures of depression

All patients completed the Beck Depression Inventory (BDI) (Hautzinger et al. 1995) and the IDS-SR_30_ (Trivedi et al. 2004). Subscore calculation of the IDS-SR_30_ included a somatic and a cognitive subscale as published by Duivis et al. (2013). The cognitive scale contains 10 IDS-SR_30_ items, one for each of the following symptoms domains: Feeling sad or irritable, the quality of mood, concentration/decision making, self-perception, suicidal ideation, general interest, as well as capacity for pleasure excluding and including sexuality. The somatic subscale includes items on sleep, appetite, weight, energy level, psychomotor retardation/restlessness, and leaden paralysis/physical energy.

### Statistics

Major depressive disorder diagnosis was established based on the M.I.N.I. (criterion). Receiver-Operating characteristics curves were created using MatLab and MedCalc software, giving an overview of sensitivity and specificity combinations for possible thresholds in each questionnaire. Error estimates and confidence intervals were calculated by bootstrapping using 1000 replications. Using MedCalc, the BDI, IDS-SR_30_ total and somatic and cognitive subscore ROC curves were compared statistically using the method of DeLong et al. (1988) for the calculation of the Standard Error of the Area Under the Curve (AUC) and of the difference between two AUCs. This algorithm is particularly useful because it adjusts the AUCs for the expected frequency of the condition (MDD in this case) in the population of interest (in this case MS). Based on available epidemiological research (Patten et al. 2003), we estimated the MDD point prevalence in the MS population at 25%. AUC values were interpreted according to the following guidelines: 0.9–1 excellent, 0.8–0.9 good, 0.7–0.8 fair, 0.6–0.7 poor.

Cutoff values were established with the (0, 1) minimum distance method giving equal weight to sensitivity and specificity. Distributions of the thresholds as well as the false-positive and false-negative rates were determined to estimate uncertainty and control for the small sample size. Finally, BDI and IDS-SR_30_ scores were correlated using Pearson correlation coefficients. All values are given as mean ± SEM. *P*-values of <0.05 were considered significant.

## Results

### Demography

Patients were aged 22–66 years old (M = 49.06 ± 1.89). About 75% of the participants were female (*n* = 25). Clinical and demographic characteristics can be found in Table 1999.

**Table 1:** Clinical and demographic characteristics. Mean (M) and standard error of the mean (SEM) for the Beck Depression Inventory (BDI), total 30-item self-report Inventory of Depressive Symptomatology (IDS-SR_30_), somatic (IDS som), and cognitive IDS subscale (IDS-SR_30_cog) as well as the Quick IDS (QIDS-SR) 16-item short version. Multiple sclerosis patients were diagnosed as depressed major depressive disorder (MDD) and nondepressed (no MDD) via structured clinical interviews (The Mini International Neuropsychiatric Interview, M.I.N.I.).

	Depressed (meeting MDD criteria M.I.N.I.) *n* = 21	Nondepressed (not meeting MDD criteria M.I.N.I) *n* = 10
Age	50.60 ± 2.15	48.41 ± 3.95
Education (years)	10.95 ± 0.34	11.10 ± 0.53
Sex (male/female)	4/17	2/8
Disease course (CIS/RRMS/SPMS/PPMS/unclear)	3/12/2/3/1	0/3/6/1/0
BDI	21.8 ± 1.90	9.6 ± 2.13
IDS-SR30	37.5 ± 10.58	21.2 ± 3.29
IDS som	8.76 ± 0.62	5.5 ± 1.26
IDS cog	13.8 ± 10.96	5.4 ± 1.23
QIDS	15.47 ± 1.39	8.67 ± 1.96

CIS, clinically isolated syndrome; PPMS, primary progressive multiple sclerosis; RRMS, relapsing-remitting multiple sclerosis; SPMS, secondary progressive multiple Sclerosis. Data are presented as mean ± SEM.

### Depression frequency and severity

Twenty-one of the 31 patients enrolled fulfilled the criteria of MDD according to M.I.N.I. interviews. As expected, most patients had also psychiatric comorbidities including other mood disorders (dysthymic disorder, *n* = 4; lifetime mania or hypomania, *n* = 5), anxiety disorders (generalized anxiety disorder, *n* = 11; agoraphobia with and without panic disorder, *n* = 5; social phobia *n* = 5, posttraumatic stress disorder, *n* = 1; OCD, *n* = 1), or substance abuse (*n* = 2).

As expected, patients with MDD scored well over usual cutoffs for clinical depression in the IDS-SR_30_ as well as the BDI (Table 1999). In addition, due to screening criteria for this patient group, IDS-SR_30_ depression scores were also slightly elevated in the patients not meeting diagnostic criteria for MDD (Table 1999). BDI and IDS-SR_30_ showed a highly significant intercorrelation (*r* = 0.82, *P* < 0.0001, 95% CI 0.67–0.91).

### ROC analyses

**Figure 1:**
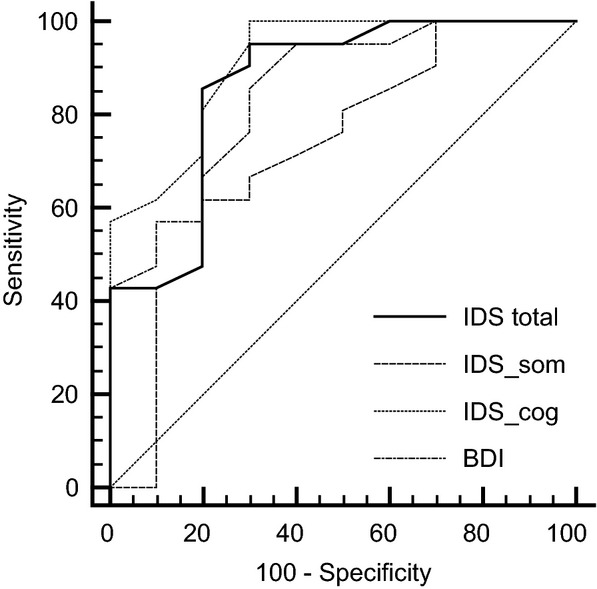
Receiver-Operating Characteristic (ROC) curves for the Beck Depression Inventory (BDI), the self-rated Inventory of Depressive Symptomatology (IDS-SR30) total score (IDS total) and the cognitive (IDS cog) and somatic (IDS som) subscores for predicting major depressive disorder based on structured clinical interviews (M.I.N.I).

All ROC-derived sensitivity and specificity values are shown in Table 2003, and ROC curves are depicted in Fig.1. The AUC derived from the ROC for the IDS-SR_30_ indicated good accuracy (AUC = 0.86 ± 0.08). A cutoff of 28 (SD (IDS-SR_30_–total) = 3.66) provides a sensitivity of 80% and specificity of 77% (Table 2003). The false-positive (negative) rate for IDS-SR_30_ total when using 28 as the cutoff was estimated as 19.9 ± 7.3% (23.0 ± 12.0%). This results in a positive likelihood ratio of 5.67 and a negative likelihood ratio of 0.38. Furthermore, we determined diagnostic accuracy of the IDS-SR_30_ cognitive and somatic subscales. The cognitive subscale reached excellent accuracy (AUC = 0.91 ± 0.06). For the IDS-SR_30_ cognitive scale, the analysis yielded a cutoff value of 10 (sd(IDS-SR_30_–cog) = 3.15, Table 2003). The false-positive (negative) rate for the cognitive IDS-SR_30_ subscale cutoff was estimated as 19.30 ± 7.74% (30.69 ± 13.32%), leading to a positive likelihood ratio of 4.25 and a negative likelihood ratio of 0.25. In contrast, the IDS-SR_30_ somatic scale only showed fair accuracy (AUC = 0.72 ± 0.1). The QIDS-SR had good accuracy AUC of 0.80 ± 0.08 (CI 0.669–0.997) with a suggested cutoff of 13 (Sensitivity 66.67, Specificity 90.00).

**Table 2:** IDS-SR_30_ sensitivity and specificity.

Cutoff	Sensitivity	95% CI	Specificity	95% CI
≥9	100.00	88.4–100.0	0.00	0.0–24.7
>15	100.00	88.4–100.0	38.46	13.9–68.4
>19	96.67	82.8–99.9	46.15	19.2–74.9
>21	96.67	82.8–99.9	61.54	31.6–86.1
>25	90.00	73.5–97.9	61.54	31.6–86.1
>26	83.33	65.3–94.4	69.23	38.6–90.9
>27	80.00	61.4–92.3	76.92	46.2–95.0
>33	53.33	34.3–71.7	76.92	46.2–95.0
>37	46.67	28.3–65.7	84.62	54.6–98.1
>38	40.00	22.7–59.4	92.31	64.0–99.8
>39	40.00	22.7–59.4	100.00	75.3–100.0
>59	0.00	0.0–11.6	100.00	75.3–100.0

Predictive value of the self-rated Inventory of Depressive Symptomatology (IDS-SR_30_) for major depressive disorder: sensitivity, specificity and their 95% confidence intervals (CI) for potential cutoff values.

**Table 3:** IDS-SR_30_ cognitive subscale sensitivity and specificity.

Cutoff	Sensitivity	95% CI	Specificity	95% CI
≥1	100.00	86.8–100.0	0.00	0.0–24.7
>4	100.00	86.8–100.0	53.85	25.1–80.8
>5	96.15	80.4–99.9	61.54	31.6–86.1
>8	92.31	74.9–99.1	61.54	31.6–86.1
>9	80.77	60.6–93.4	69.23	38.6–90.9
>10	69.23	48.2–85.7	69.23	38.6–90.9
>11	53.85	33.4–73.4	76.92	46.2–95.0
>12	50.00	29.9–70.1	92.31	64.0–99.8
>14	34.62	17.2–55.7	92.31	64.0–99.8
>15	30.77	14.3–51.8	100.00	75.3–100.0
>24	0.00	0.0–13.2	100.00	75.3–100.0

Predictive value of the self-rated cognitive Inventory of Depressive Symptomatology subscale for major depressive disorder: sensitivity, specificity, and their 95% confidence intervals (CI) for potential cutoff values.

Receiver-Operating characteristics analysis for the BDI revealed good accuracy (AUC = 0.85 ± 0.07) and a cutoff value of 12 (SD (BDI) = 3.69, Table 2002). This cutoff yields Sensitivity of 88% and Specificity of 70%. The false-positive (negative) rate for the BDI with this cutoff was estimated as 12.48 ± 6.72% (30.15 ± 15.03%). For the BDI, we thus determined a positive likelihood ratio of 6.00 and a negative likelihood ratio of 0.43.

**Table 4:** BDI sensitivity and specificity.

Cutoff	Sensitivity	95% CI	Specificity	95% CI
≥0	100.00	85.8–100.0	0.00	0.0–30.8
>5	100.00	85.8–100.0	30.00	6.7–65.2
>7	95.83	78.9–99.9	40.00	12.2–73.8
>8	95.83	78.9–99.9	60.00	26.2–87.8
>11	87.50	67.6–97.3	70.00	34.8–93.3
>13	79.17	57.8–92.9	70.00	34.8–93.3
>14	70.83	48.9–87.4	80.00	44.4–97.5
>17	58.33	36.6–77.9	80.00	44.4–97.5
>19	58.33	36.6–77.9	90.00	55.5–99.7
>20	50.00	29.1–70.9	90.00	55.5–99.7
>21	45.83	25.6–67.2	100.00	69.2–100.0
>44	0.00	0.0–14.2	100.00	69.2–100.0

Predictive value of the Beck Depression Inventory (BDI) for major depressive disorder: sensitivity, specificity, and their 95% confidence intervals (CI) for potential cutoff values.

Comparison of AUC values for the IDS-SR_30_ total score, IDS-SR_30_ cognitive subscore, IDS-SR_30_ subscale and BDI yielded significant differences between IDS-SR_30_ total and IDS-SR_30_ somatic (*P* = 0.02) as well as IDS-SR_30_ cognitive and IDS-SR_30_ somatic (*P* = 0.04) while the difference between the BDI and IDS-SR_30_ somatic subscore failed to reach statistical significance (*P* = 0.09). There were no significant differences between the IDS-SR_30_ total score and the cognitive subscore (*P* = 0.38) as well as the BDI (*P* = 0.80).

## Discussion

Our results indicate that two widely used patient-based instruments, the IDS-SR_30_ and the BDI, yield good accuracy for depression in MS when compared to a structured clinical interview. Moreover, we provide first evidence for validity of the IDS-SR_30_ total score, IDS-SR_30_ cognitive subscale, and the QIDS-SR short from for assessment of depression in MS.

Several studies have previously investigated psychometric properties of self-report depression questionnaires in MS. For the most part, analyses have been restricted to measures of reliability (such as internal consistency), correlational analyses with questionnaires measuring related concepts, or response to therapy (Nyenhuis et al. 1995; Sullivan et al. 1995; Avasarala et al. 2003; Benedict et al. 2003; Moran and Mohr 2005; Mohr et al. 2007; Honarmand and Feinstein 2009; Quaranta et al. 2012; Wang and Gorenstein 2013). However, a few have assessed diagnostic accuracy against a structured clinical interview: Mohr et al. (2007) demonstrated that two questions covering the two core symptoms of MDD (anhedonia and depressed mood) yield 99% sensitivity and 87% specificity. This approach is, therefore, highly accurate as a screening tool, although a more recent study reported lower estimates of specificity and sensitivity for this instrument (Patten et al. 2015). Moreover, it does not provide a quantitative score of depression severity. The 8-item depression subscale of the HADS (Honarmand and Feinstein 2009) was previously found to provide a sensitivity of 90% and a specificity of 87% for MDD in MS (as determined by the SCID). In this study, the authors also conducted a ROC analysis, which yielded an AUC of 0.94, which can be considered excellent. A recent study explored the diagnostic accuracy of the BDI in Italian MS patients against the SCID (Quaranta et al. 2012). Here, the AUC was 0.83 (good accuracy). The results from our study confirm the good accuracy of the BDI (AUC = 0.85), although we obtained markedly better sensitivity. We also provide first evidence that a comparatively new depression questionnaire, the IDS-SR_30_, also provides good accuracy when validated against a structured clinical interview.

The very recent study by Patten and colleagues provided the first available head-to-head comparison of self-report scales of depression in MS (Patten et al. 2015) and showed good accuracy for the CES-D, the PHQ (9 and 2), and the HADS. Since the PHQ is available free of charge, it might therefore be particularly interesting. With our study, there is now another freely available instrument (IDS) available for screening in MS depression. Moreover, our results also provide a direct comparison to the BDI, the only instrument that reached a sufficient level of evidence in the AAN guidelines.

Taken together, there are now several reliable and valid strategies for interested researchers and clinicians to screen for and quantify depression in MS, each with specific advantages and disadvantages. All scales evaluated to date (BDI, IDS-SR_30_, HADS, 2-question screen, PHQ, CES-D) show good sensitivity and specificity around 80% or higher. The QIDS-SR, however, appears to be less sensitive but more specific. As noted in the AAN guidelines (Minden et al. 2014), “valid screening tools might improve identification of individuals who could benefit from further evaluation and treatment.” If this is the goal, a low false-negative rate is required. In our study, the IDS-SR_30_ had a markedly better false-negative rate (23%) compared to the BDI (30%). However, this still means that 23% of cases will be missed.

Clinically, a high false positive rate is less of a concern; it does however increase the administrative burden and may waste resources in particular settings such as primary care. For maximum sensitivity, specificity, and cost-effectiveness, the two-question approach proposed by David Mohr and colleagues might be the ideal choice. However, it does not yield a quantitative score of depression severity, which may be necessary in a research setting or to monitor treatment response in clinical care. The HADS provides a middle ground of a comparatively short scale offering both good accuracy for MDD diagnosis as well as a quantitative score. Generally, the HADS is a good measure for symptom severity in somatic, psychiatric, primary care patients and in the general population (Bjelland et al. 2002) and is therefore widely used. However, more recent work has revealed that it lacks consistent differentiation between symptoms of anxiety and depression (Cosco et al. 2012) and it does not cover all symptom domains of MDD.

The IDS-SR_30,_ validated for the first time in MS patients in the current report, in our opinion has a number of features that make it a good option for measuring depression in MS: (1) it covers all DSM-IV criteria (and only those) (2) it offers parallel patient- and clinician-rated versions; (3) it was translated in many languages and is increasingly used; and (4) subscales for cognitive and somatic symptoms can be constructed (Duivis et al. 2013) as we have done in our present analysis and an algorithm for identification of DSM-assigned melancholic depression based on the items of the IDS-SR_30_ is available (Khan et al. 2006). This might be particularly relevant for use in studies to explore novel biological substrates of depression in MS as these were found to differ between data-driven designations of melancholic and atypical idiopathic depression (Lamers et al. 2013). Similar dissociations between biological correlates and clinical features might also exist in MS-associated depression, as our previous research has indicated that affective and cognitive symptoms of depression in MS might be more closely related to neuroendocrine-limbic abnormalities (Gold et al. 2010, 2014) while vegetative/somatic aspects show closer correlations with markers of inflammation (Gold et al. 2011).

First applications in an RCT for a behavioral intervention (exercise) in MS also suggest that the IDS-SR_30_ may be responsive to detect changes in depressive symptomatology (Briken et al. 2014). Sensitivity to change remains an important issue for depression questionnaires in MS that have not systematically been addressed.

Some limitations have to be considered when interpreting the results from our present study. First of all, the sample size was small and all our patients were contacted because they had previously shown elevated depressive symptoms, that is, the sample was preselected for elevated levels of depression. On one hand, this sample might be a good model for clinical situations where accurate diagnosis is particularly important. On the other hand, in larger samples including many patients with very low or no depressive symptoms, diagnostic accuracy of IDS-SR_30_ and BDI may be higher than reported here.

Despite finding the IDS-SR_30_ somatic subscale to show only fair accuracy, the total IDS score was not found to perform significantly worse than the IDS-SR_30_ cognitive subscale. This indicates that, while removal of somatic symptoms may be preferable, we found no evidence to suggest that it is strictly necessary for somatic symptoms to be removed from the IDS for diagnostic accuracy in MS. Future studies performed with a larger sample size will provide accurate/reliable estimates of the cutoff values. However, the specific values of the threshold estimates are not the most important results arising from this study. A far more meaningful and important result is the ability to provide estimates of the false-positive/negative rates for the various scores, given a particular score threshold. For example, we estimate the false-positive (negative) rate for IDS-SR_30_–total as 19.9 ± 7.3% (23.0 ± 12.0%), noting that the provision of error estimates implicitly accounts for the small sample size. Pragmatically, these results are perhaps the most important results in the article, as they provide an estimate of the error rates that would be expected, should the particular cutoff value (in this case, 28) be used as the decision-making criterion.

Furthermore, the present study does not address the ability of the BDI or the IDS-SR_30_ for differential diagnosis of MDD versus other affective disorders. In our sample, two patients with high scores on the BDI and IDS-SR_30_ were found who did not meet diagnostic criteria for MDD according to the M.I.N.I. When looking at the M.I.N.I. data of these individuals, we observed that both met diagnostic criteria of dysthymia. This means that while the questionnaires have readily identified a mood disorder, they do not seem to be a means of distinguishing between MDD and dysthymia. This illustrates that distinction between *different affective disorders* may therefore be a particular challenge in MS that requires clinical interviews and cannot be achieved with general self-report questionnaires for depression.

In conclusion, both the IDS-SR_30_ and the BDI are valid measures to quantify depressive symptoms and show good diagnostic accuracy. The IDS-SR_30_ cognitive subscale may be useful as a screening tool and to quantify affective/cognitive depressive symptomatology.
